# Transcriptome analysis reveals the differential inflammatory effects between propofol and sevoflurane during lung cancer resection: a randomized pilot study

**DOI:** 10.1186/s12957-023-02891-4

**Published:** 2023-01-16

**Authors:** Sufang Wang, Mengjiao Li, Suna Cai, Wei Zhang

**Affiliations:** 1 School of Life Sciences, Northwestern Polytechnical University, Xi’an, 710072 Shaanxi China; 2 Department of Anesthesiology and Perioperative Medicine, People’s Hospital of Zhengzhou University, Henan Provincial People’s Hospital, Zhengzhou, 450003 Henan China

**Keywords:** Inflammatory effects, Lung cancer, RNA-seq, Propofol, Sevoflurane

## Abstract

**Background:**

Propofol and sevoflurane are two commonly used perioperative anesthetics. Some studies have found that these anesthetic drugs affect tumorigenesis. Previous studies have mostly focused on in vitro experiments, and the specimens collected were mainly peripheral body fluids, lacking direct evidence of the impact of anesthetic drugs on human tissues. This study aimed to elucidate the effects of propofol and sevoflurane on lung cancer using next-generation sequencing through an in vivo experiment.

**Methods:**

Patients were randomly assigned to a group receiving either propofol or sevoflurane during surgery. Then, the patients’ tumor and paired normal samples were collected and sequenced by next-generation sequencing. Differentially expressed genes (DEG) were analyzed by two statistical models, followed by cluster analysis, PCA, Gene Ontology, and KEGG pathway analysis. Candidate genes were confirmed by qRT–PCR.

**Results:**

The demographic data of the two study groups were not statistically significant. Through single-factor model analysis, 810 DEG in the propofol group and 508 DEG in the sevoflurane group were obtained. To better reflect the differential effects between propofol and sevoflurane while reducing the false-positive DEG, we used multifactor model analysis, which resulted in 124 DEG. In PCA and cluster analysis, four groups (propofol cancer group, propofol normal group, sevoflurane cancer group, sevoflurane normal group) were separated adequately, indicating the accuracy of the analysis. We chose seven significant pathways (cellular response to interleukin-1, chemokine-mediated signaling pathway, chemokine signaling pathway, cytokine–cytokine receptor interaction, inflammatory response, immune response, and TNF signaling pathway) for downstream analysis. Based on the pathway analysis, three candidate genes (CXCR1, CXCL8, and TNFAIP3) were chosen, and their qRT–PCR results were consistent with the sequencing results.

**Conclusions:**

Through RNA-seq analysis, the effects of propofol and sevoflurane during lung cancer resection were different, mainly in inflammatory-related pathways, which might be possibly by targeting CXCL8.

**Trial registration:**

Trial registry number was ChiCTR1900026213.

**Supplementary Information:**

The online version contains supplementary material available at 10.1186/s12957-023-02891-4.

## Introduction

Lung cancer is the leading cause of cancer-associated mortality worldwide [1]. Nearly 80% of 15 million cancer patients will undergo surgery and require anesthesia [2]. Propofol and sevoflurane are the two most commonly used anesthetic agents in surgery. However, it has been shown that anesthetic drugs may affect tumorigenesis [3–8].

Several in vivo studies have investigated the effects of propofol and sevoflurane on cancer. For example, among patients who received propofol during lung cancer resection, their perioperative inflammatory responses and rates of intraoperative adverse reactions were significantly reduced compared with those who received sevoflurane [9]. Another similar study showed that thoracic paravertebral nerve block-propofol anesthesia could reduce the serum concentration of vascular endothelial growth factor (VEGF) [10]. A recent pilot study found differentially expressed microRNAs in circulating extracellular vesicles among patients with colorectal cancer, which showed that propofol may have an inhibitory effect on cell proliferation and migration and enhance tumor cell apoptosis [7]. One retrospective study revealed that propofol-based total intravenous anesthesia had a better overall survival rate than volatile anesthesia [11]. However, another study found that the effects of anesthetic drugs were minimal in terms of immune activity during breast cancer surgery [12]. Similar conclusions were made that there were no significant differences in the overall and recurrence-free survival rates between the volatile anesthesia group and the propofol-based anesthesia group in a nationwide retrospective cohort study [13]. Taken together, the effects of anesthetic drugs on cancer are still controversial.

Researchers also evaluated the effects of propofol and sevoflurane on lung cancer in vitro. It has been demonstrated that propofol has an adverse effect on cell viability and promotes apoptosis by downregulating the miR-21-5p/MAPK10 axis in the non-small cell lung cancer cell lines A549 and H1299 [5]. Similar results have been observed in which propofol inhibited cell growth and migration and promoted apoptosis of A549 cells [8, 14–16]. However, some studies have reached different conclusions: sevoflurane affects apoptosis in A549 cells [17] and suppresses metastasis of A549 cells by modulating hypoxia-inducible factor-1α [18].

Overall, the effects of propofol and sevoflurane on patients who undergo lung cancer surgery are still unclear. More importantly, the underlying molecular mechanism remains unexplored. In this research, we conducted an in vivo study, and eight lung cancer patients with tumor and paired normal samples were collected, among which 4 patients received propofol and 4 patients received sevoflurane. We used RNA-seq to obtain a comprehensive profile of the differentially expressed genes (DEG), aiming to decipher the key factors of propofol and sevoflurane on lung cancer. Finally, selected candidate genes were confirmed by qRT–PCR. This study sheds light on the effects of anesthesia at the whole transcriptome level and explores the possible genes and pathways involved in tumorigenesis, which provides a basis for future work.

## Materials and methods

### Ethics approval and consent to participate

This prospective study was approved by the Ethics Committee of Henan Provincial People’s Hospital (2019-lushen-41). The trial registry number was ChiCTR1900026213, and it was registered on September 26, 2019 (http://www.chictr.org.cn/showproj.aspx?proj=43733). Written informed consent and information release approvals were obtained from all patients prior to their participation in this study. The study protocol complied with the 1975 Declaration of Helsinki.

### Participant selection

This study was designed as a randomized, single-blind study. We enrolled 28 patients who underwent lung cancer surgery, aged 18–65 years, had a BMI of 18–25 kg/m^2^, and had an ASA status of 1–3 from Oct 2019 to May 2020.

#### Inclusion criteria

These are no history of blood disease or other metabolic disorders, no history of hormone use, no autoimmune disease, and no history of radiotherapy, chemotherapy, or immunotherapy.

#### Exclusion criteria

These are history of other operations, refusal to participate in the trial, drop-out from the trial, data loss, and severe hypoxemia during surgery (SpO_2_ below 90% over 1 min after F_i_O_2_ is adjusted to 100%).

##### Rejection criteria

The are changes in surgical procedure, blood transfusion, postoperative diagnosis confirmed not adenocarcinoma by an independent pathological, and one-lung ventilation (OLV) duration less than 1 h.

Patients were randomly allocated to two groups according to a computer-generated random number table: the propofol group (P group) and the sevoflurane group (S group). After intravenous injection of 0.05 mg/kg midazolam, 0.3–0.5 μg/kg sufentanil, 0.2–0.3 mg/kg etomidate, and 0.6–0.9 mg/kg rocuronium were administered for induction, and a double-lumen bronchial tube was inserted. Mechanical ventilation was performed after induction: F_i_O_2_ 70%, *VT* 6–8 ml/kg, *RR* 10–14 times/min, and I:E 1:2. During one-lung ventilation, the RR was 12–16 times/min, and the F_i_O_2_ was 70%. The other parameters remained unchanged, and P_ET_CO_2_ was maintained at 35–45 mmHg.

Anesthesia maintenance was achieved by propofol and sevoflurane in the P group and S group, respectively, combined with remifentanil and intermittent intravenous infusion of cisatracurium. The BIS was maintained between 40 and 50, and the fluctuation ranges of the HR and MAP were maintained at no more than 20% of the baseline values. The intraoperative intravenous infusion of Ringer’s solution of sodium lactate was 2–3 ml/kg/h. Oxycodone (1 mg/kg) was used for postoperative patient pain control.

### Sample collection

Once the lung specimens were harvested, samples of tumor tissue and normal tissue (that was at least 5 cm away from the tumor tissue) were immediately collected. After washing with PBS and drying with filter paper, the specimens, soaked in RNA preservation solution, were placed at −80 °C until use. To ensure that the tumor tissue and the normal tissue were harvested correctly, the lung specimens were verified by an independent pathologist.

### RNA extraction and sequencing library construction

Total RNA was extracted following the manufacturer’s protocol. The libraries were sequenced on the Illumina sequencing platform (HiSeq™ 2500), and 150 bp paired-end reads were generated.

### Quality control of raw reads and alignment to the reference genome

Trimmomatic [19] was used for trimming the raw reads, including removing the adapter sequences, low-quality bases, and reads. After obtaining the clean reads, HISAT2 [20] with default parameters was used to align the clean reads to the reference genome (version GRCH38).

### Differentially expressed genes (DEG) analysis

The DESeq2 package [21] was used to identify DEG.

1. Single-factor model: Identifying DEG between cancer and normal samples within each group (P group or S group):
  $${Y}_{ij}=\mu +{\alpha}_i+{\varepsilon}_{ij}$$

where i is the condition (cancer or normal) and j is the replicates.

2) Multifactor model: Identifying DEG across all four conditions:
  $${Y}_{ijk}=\mu +{\alpha}_i+{\beta}_j+{\varepsilon}_{ijk}$$

where i is factor 1 (P group or S group), j is factor 2 (cancer or normal), and k is the replicates.

### Other bioinformatics analyses

Cluster analysis and principal component analysis were performed using R software. Gene Ontology enrichment and KEGG pathway analysis were performed using DAVID (https://david.ncifcrf.gov) [22].

### Quantitative real-time PCR (qRT–PCR) analysis

The total RNA of tissues was efficiently extracted with the EasyPure® RNA Purification Kit (Transgen Biotech, ER701-01, Beijing, China). The obtained total RNAs were used for first-strand cDNA synthesis, and their relative expression was estimated by the q-PCR method (TransStart® Top Green qPCR SuperMix, Transgen Biotech, AQ131-01, Beijing, China) on a CFX96 Touch qPCR System (Bio-Rad Laboratories, Hercules, CA, USA). β-actin was used as an internal reference gene, and the 2^−ΔΔCt^ method was employed to calculate the relative expression ratio of the candidate genes. The specific primers are listed in supplemental Table S1.

### Statistical analysis

Data from the qRT–PCR experiments are expressed as the mean ± SE of at least three independent experiments (*n* ≥ 3). The *P*-value was calculated by a two-sided Student’s *t*-test. Categorical data analysis of the demographics was calculated by the chi-square test.

## Results

### Basic information of the study groups

**Fig. 1:**
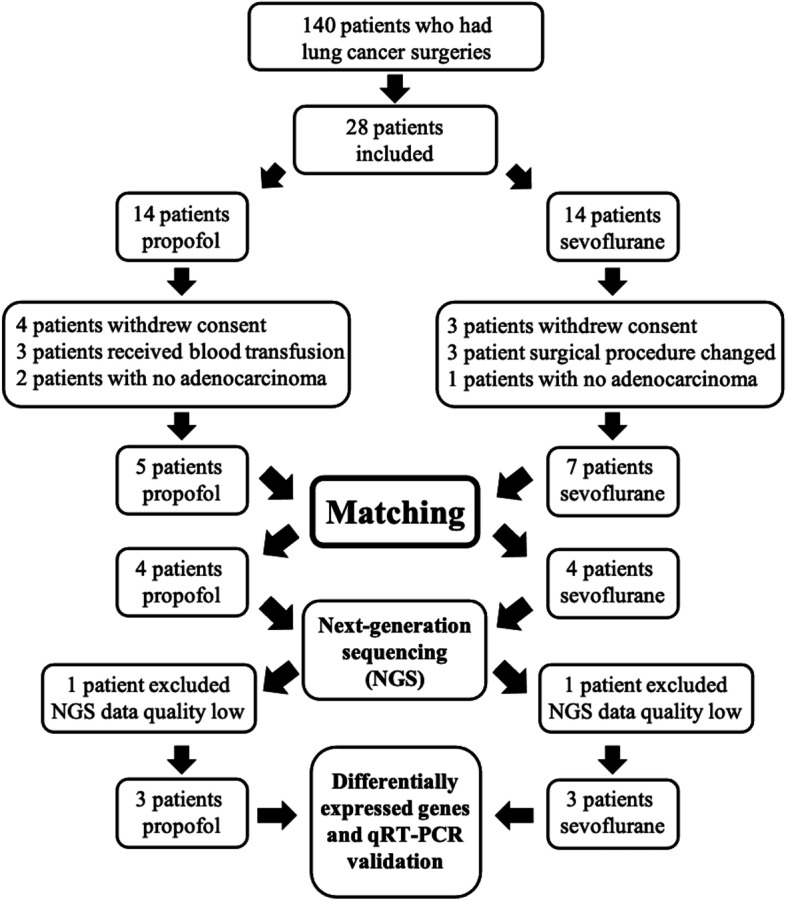
Flowchart of the patient selection, matching process, and next-generation sequencing (NGS) in this research

We collected the study groups’ (propofol group and sevoflurane group) demographic data, including age, sex, body mass index, ASA physical status, smoking history, diabetes type 2, arterial hypertension, coronary artery disease, UICC tumor stage, duration of surgery, and duration of one-lung ventilation (Table 1). None of these variables was significantly different between the groups (*P*-value > 0.05). To explore the effects of propofol (P) and sevoflurane (S) during lung cancer resection, we further matched the two groups and used RNA-seq to reveal the molecular mechanism (Fig. 1).

**Table 1:** Characteristics of demographic data between propofol and sevoflurane groups

Parameter	Propofol	Sevoflurane	***p***-value
Age (yr)^a^	55–70	42–71	0.49
Gender (male/female)	3/2	2/5	0.28
Body mass index (kg/m^2^)^a^	24.8–29.7	21.5–30.1	0.36
ASA physical status (II/III)	3/2	5/2	0.68
Smoke history (yes/no)	3/2	2/5	0.28
Diabetes type2 (yes/no)	2/3	0/7	0.07
Arterial hypertension (yes/no)	1/4	1/6	0.79
Coronary artery disease (yes/no)	0/5	1/6	0.38
UICC tumor stage (Ia/Ib/IIa/IIb/IIIa/IIIb)	3/0/0/1/0/1	6/1/0/0/0/0	0.29
Duration of surgery (min)^a^	150–240	160–225	0.25
Duration of one-lung ventilation (min)^a^	120–210	130–195	0.21

^a^Data are the range (smallest to largest)

### Quality of NGS and mapping rate

**Fig. 2:**
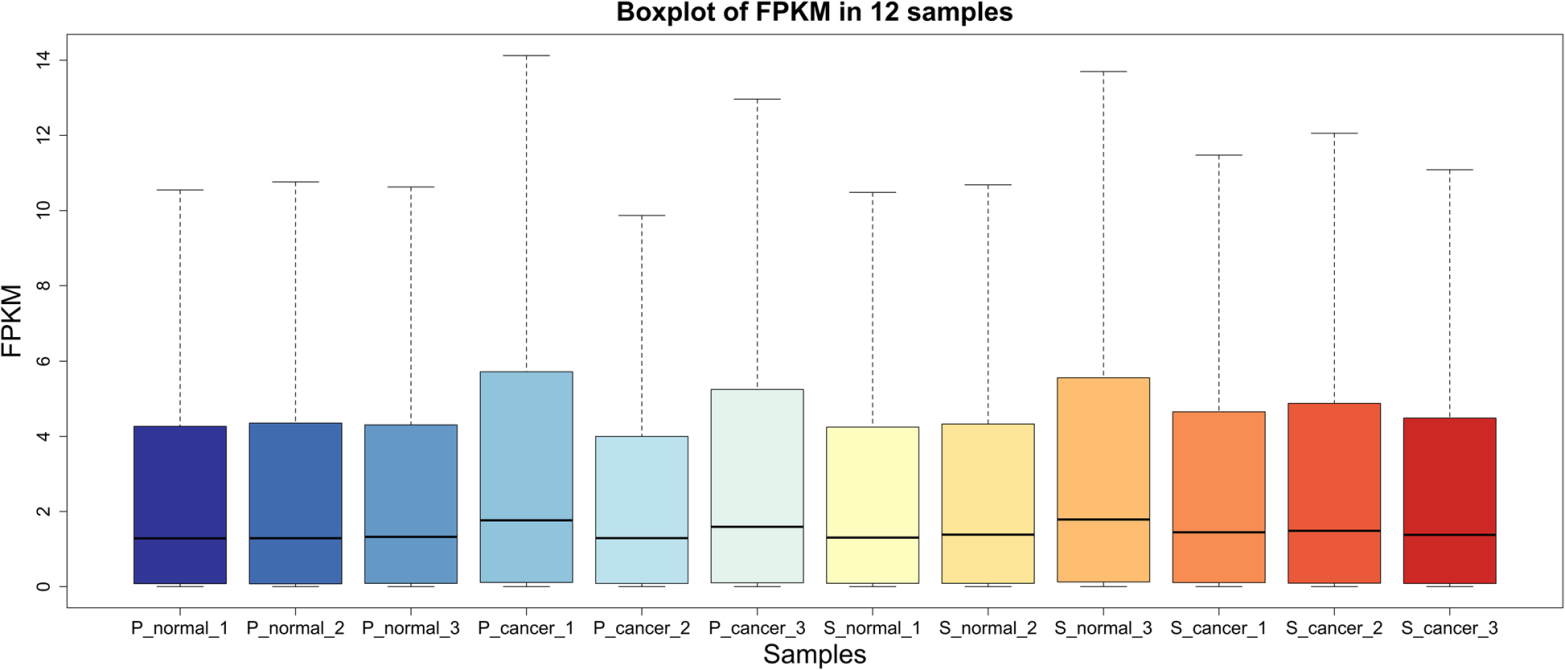
Boxplot of fragments per kilobase per million (FPKM) in each sample

We sequenced a total of 16 samples, 4 patients in each anesthesia group (P or S group) with tumor and paired normal samples. However, 2 sets of patient data (one from the P group, one from the S group) did not pass the quality control, which resulted in 12 samples (sequence libraries) remaining. After sequencing, we obtained a total number of 170.05 GB clean reads with an average of 14.17 GB of data for each library. The sequence quality score Q30 ranged from 92.58 to 93.92%, and the average GC content was 47.24% (Table 2). Then, we mapped the clean reads to the human reference genome. The total mapping rate ranged from 96.87 to 97.59%, and the uniquely mapped rate was 89.85 to 92.56% (Table 2). The distributions of fragments per kilobase per million (FPKM) in all 12 samples were consistent, indicating good sequencing quality (Fig. 2).

**Table 2:** Sequencing quality and mapping rate in each sample

Sample	Raw data	Clean data	Q30	GC content	Total mapping rate	Uniquely mapped rate
P_cancer_1	14.86G	13.21G	93.19%	47.81%	96.87%	89.85%
P_cancer_2	15.57G	13.59G	92.82%	46.71%	96.72%	90.48%
P_cancer_3	16.73G	14.95G	93.26%	47.03%	97.11%	91.29%
P_normal_1	15.75G	13.68G	92.58%	46.51%	97.17%	92.56%
P_normal_2	17.41G	15.32G	93.02%	46.35%	97.24%	92.31%
P_normal_3	15.08G	12.90G	93.56%	47.58%	97.38%	92.17%
S_cancer_1	17.60G	15.67G	93.73%	47.40%	97.45%	90.80%
S_cancer_2	15.38G	13.56G	93.80%	47.46%	97.12%	90.55%
S_cancer_3	15.26G	13.54G	93.79%	47.83%	97.06%	90.39%
S_normal_1	16.17G	14.27G	93.85%	46.58%	97.64%	92.35%
S_normal_2	16.39G	14.40G	93.91%	47.44%	97.45%	90.99%
S_normal_3	16.84G	14.96G	93.92%	48.23%	97.59%	90.70%

### Differentially expressed genes (DEG) identified by a single factor model

We first performed differential gene analysis within each study group to (1) compare the P group cancer samples vs. the P group normal samples, aiming to discover the effect of propofol on lung cancer and (2) compare the S group cancer samples vs. the S group normal samples, aiming to discover the effect of sevoflurane on lung cancer. The DEG threshold was defined as *FDR* ≤ 0.01 and log_2_FC ≥ ± 2. As a result, we obtained 810 DEG in the P group and 508 DEG in the S group.

**Fig. 3:**
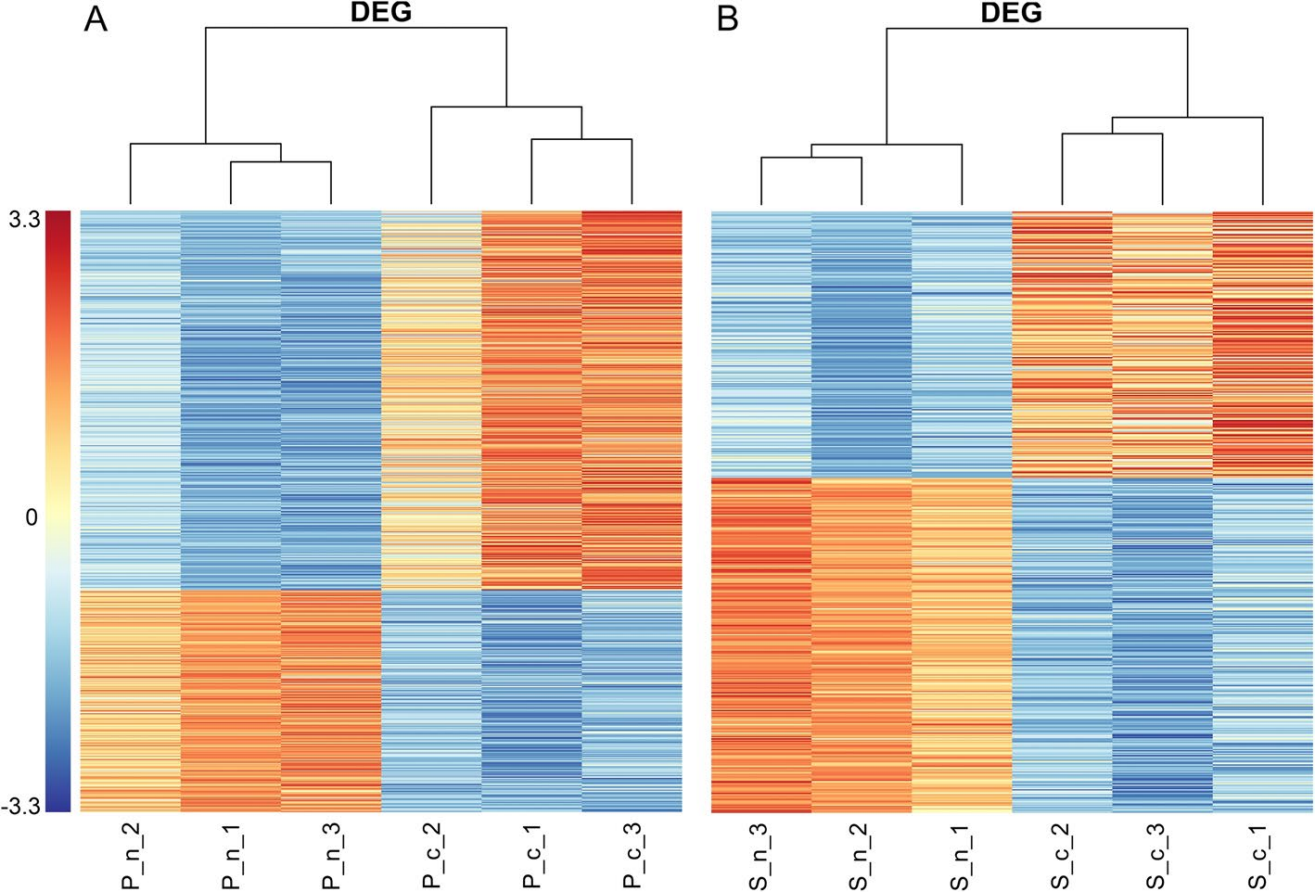
Heatmap of cluster analysis using differentially expressed genes (DEG) between normal samples (*n*) and cancer samples (c). **A** Heatmap of DEG from paired P group comparison. **B** Heatmap of DEG from paired S group comparison

We used the hierarchical cluster method to cluster the DEG. From the heat map, there were clearly two clusters in each group (Fig. 3 A and B); all cancer samples were in one cluster, and the normal samples were in another. We further compared these two sets of DEG and found 191 DEG in both groups. Then, we wondered whether these 191 DEG could reveal the different effects of propofol and sevoflurane on cancer. However, from a statistical perspective, simply looking at the overlap of DEG in two comparisons is not sufficient because too many false-positives will be produced. If we directly compared P cancer samples vs. S cancer samples, this was also not a good comparison for two reasons: (1) the control group was not the same and (2) the heterogeneity of the patients. Therefore, an advanced analysis was needed.

### DEG identified by a multifactor model

We developed an integrated statistical model to identify DEG, which takes the information of the anesthesia drugs (propofol and sevoflurane) and the conditions (cancer and normal) into account (see the details in the “Materials and methods”). The threshold was set as *FDR* ≤ 0.05 and log_2_FC ≥ ± 1. As a result, 124 DEG were identified that responded not only to the condition (normal or cancer) but also to the effect of propofol and sevoflurane. Among these DEG, 53 DEG were downregulated, and 71 DEG were upregulated.

**Fig. 4:**
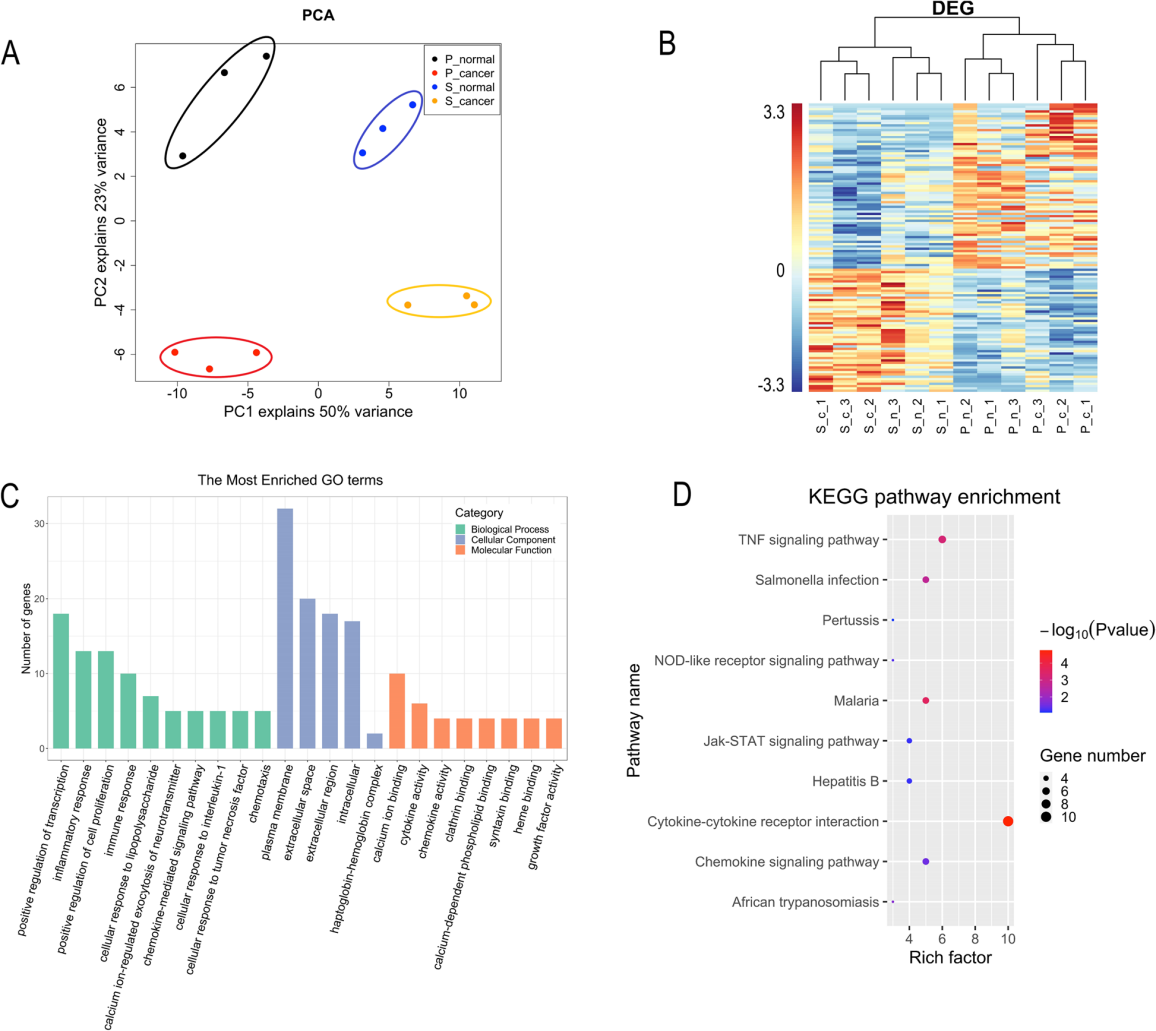
RNA-seq analysis on 124 differentially expressed genes (DEG). **A** Principal component analysis (PCA). **B** Heatmap of cluster analysis. **C** Bar graph of GO enrichment analysis. **D** Bubble gram of KEGG pathway analysis

After obtaining this comprehensive list of DEG, we performed a principal component analysis (PCA), a statistical procedure to transform data from high dimensions into low dimensions. This type of plot is useful for visualizing the overall effect of experimental conditions. In the PCA figure, four groups (P cancer group, P normal group, S cancer group, S normal group) were separated adequately (Fig. 4A). For example, the P group and S group separated very well from principal component 1 (PC1), and the cancer group and normal group separated very well from principal component 2 (PC2). Overall, PC1 can explain 50% of the variance, and PC2 can explain 23% of the variance.

We also performed a hierarchical cluster analysis (Fig. 4B). From the heatmap, the P group and S group were clearly divided into two clusters; moreover, the cancer and normal conditions were also separated well in each group, indicating the accuracy of the integrated statistical model. Taken together, these results suggested that the effects of propofol and sevoflurane were different.

### Gene Ontology (GO) and KEGG pathway analysis

GO enrichment analysis and KEGG pathway analysis were performed using 124 DEG. The threshold was taken as a *P*-value < 0.01. The GO analysis consists of three parts as follows: biological process, cellular component, and molecular function. Terms that were significant in each part and the number of genes in each process were plotted as a bar graph (Fig. 4C). The biological process was the most interesting and informative one, and the top 10 significant terms in the biological process category were (1) positive regulation of transcription, (2) inflammatory response, (3) positive regulation of cell proliferation, (4) immune response, (5) cellular response to lipopolysaccharide, (6) calcium ion-regulated exocytosis of neurotransmitter, (7) chemokine-mediated signaling pathway, (8) cellular response to interleukin-1, (9) cellular response to tumor necrosis factor, and (10) chemotaxis.

In the KEGG pathway analysis, the top 10 significant pathways with their *P*-values, and the number of genes involved in each pathway was plotted in a bubble diagram (Fig. 4D), including (1) cytokine–cytokine receptor interaction, (2) TNF signaling pathway, (3) malaria, (4) *Salmonella* infection, (5) chemokine signaling pathway, (6) hepatitis B, (7) Jak-STAT signaling pathway, (8) African trypanosomiasis, (9) NOD-like receptor signaling pathway, and (10) pertussis.

From the GO and KEGG pathway analyses, we compared and integrated the significant processes and then chose to focus on seven pathways for downstream analysis (cellular response to interleukin-1, chemokine-mediated signaling pathway, chemokine signaling pathway, cytokine–cytokine receptor interaction, inflammatory response, immune response, and TNF signaling pathway). Genes involved in these pathways were also extracted (Table 3). Several genes were involved in more than one pathway, indicating they had important roles in network regulation.

**Table 3:** Pathways used for downstream analysis

Pathway	***p***-Value	Genes
Cellular response to interleukin-1	1.17E-03	**CCL20**, **CXCL8**, ZC3H12A
Chemokine-mediated signaling pathway	1.17E-03	**CCL20**, **CXCR1**, **CXCL8**
Chemokine signaling pathway	3.03E-02	**CCL20**, **CXCR1**, **CXCL8**
Cytokine-cytokine receptor interaction	1.77E-05	**CCL20**, **CXCR1**, **CXCL8**, CSF3, IL12RB2, IL18RAP, LIF, OSM
Inflammatory response	6.43E-06	**CCL20**, **CXCR1**, **CXCL8**, CYP26B1, SELE, **TNFAIP3**, ZC3H12A
Immune response	1.68E-03	**CCL20**, **CXCL8**, CSF3, FCAR
TNF signaling pathway	5.56E-04	**CCL20**, FOS, LIF, SELE, **TNFAIP3**

Genes in bold font are used for qRT–PCR analysis

### Candidate genes confirmed by qRT–PCR

**Fig. 5:**
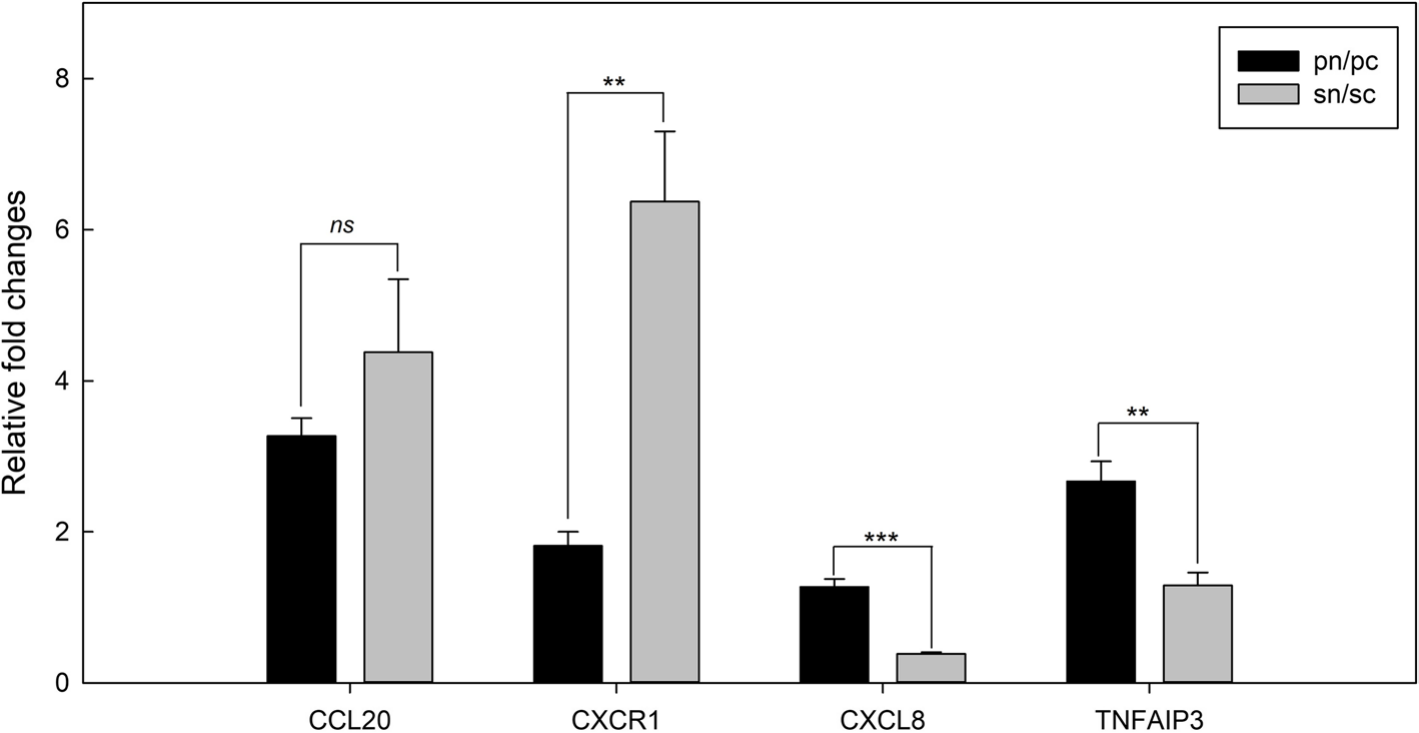
Verification of gene expression in qRT–PCR experiments. Data were expressed as mean ± SE. Significance level was indicated as *P*-value < 0.05, as *P-value < 0.005, as **P-value < 0.0005, and as ***non-significant as ns. Fold change was calculated from 2^−ΔΔCt^ values between normal (n) samples and cancer (c) samples

We chose four genes (CCL20, CXCR1, CXCL8, and TNFAIP3) for validation. To better reflect the differences between P normal (Pn), P cancer (Pc), S normal (Sn) and S cancer (Sc), we calculated the ratio of the normal samples to the cancer samples (Pn/Pc and Sn/Sc). On qRT–PCR, three genes (CXCR1, CXCL8, and TNFAIP3) were consistent with the RNA-seq results and were significantly different between P and S (Fig. 5), whereas CCL20 showed no significant difference between P and S. Both CXCR1 and TNFAIP3 showed decreased expression in the cancer samples; however, CXCR1 was more depressed under S, and TNFAIP3 was more depressed under P, indicating different levels of responses to anesthetic drugs. CXCL8 showed increased expression in S cancer samples but decreased expression in P cancer samples, which may suggest that cancer cells respond differently to P and S during lung cancer resection.

## Discussion

In this study, we aimed to elucidate the effects of propofol and sevoflurane on lung cancer. Previous studies on the relationship between anesthetic drugs and tumors have mostly focused on in vitro experiments using cell lines and in vivo experiments using animals. Studies on human subjects were mostly retrospective and observational and lacked large-scale prospective studies. Moreover, the specimens collected were primarily peripheral body fluid, lacking direct evidence of the impact of anesthetic drugs on human tumor tissues. To the best of our knowledge, this is the first in vivo study using normal human lung and cancer tissues to investigate the effects of propofol and sevoflurane on lung cancer. We used RNA-seq analysis to identify key factors, applying a more accurate and integrated statistical model to identify DEG. This improved statistical model better reflected the differential effects of P and S during lung cancer resection.

Inflammatory cytokines, growth factors, and chemokines are rich in the tumor microenvironment, facilitating tumor growth and progression [23]. Among these factors, CXCL8, a member of the CXL family, plays a crucial role in tumorigenesis and angiogenesis [24]. Accumulated studies have suggested that CXCL8 can recruit and activate immune cells in the proinflammatory environment [25]. CXCL8 mainly functions through its interactions with CXCR1 and CXCR2, and CXCL8/CXCR1 contributes to the proliferation of tumor cells [23, 26]. Therefore, the expression level of CXCL8 can be used as an indicator of tumor prognosis [27]. In our study, the results suggested that CXCL8 showed increased expression when treated with S but decreased expression when treated with P. This may suggest that P could reduce inflammation, while S could enhance inflammation.

As a G protein-coupled receptor (GPCR), CXCR1 is considered to be the main receptor for CXCL8 involved in tumorigenesis [28]. The interaction between CXCR1 and CXCL8 is mediated via the N-terminal *β*-strand of CXCR1 [29]. Recently, CXCL1 was detected on myeloid-derived suppressor cells derived from tumors [26]. Therefore, CXCL8 can recruit suppressor cells to the tumor microenvironment by recognizing CXCL1. This process may inhibit the antitumor effect of immune cells, including T cells and NK cells. Some clinical grade inhibitors, such as reparixin and CXCL1-antibody, were applied to block the binding of CXCL8 and CXCL1, expecting they may enhance the antitumor effect [23]. In this study, we found that CXCR1 showed decreased expression in cancer samples, indicating that P and S have similar functions on CXCR1.

Tumor necrosis factor alpha-induced protein 3 (TNFAIP3) is an important deubiquitinating enzyme that has an impact on tumorigenesis, immune responses, and inflammation [30, 31]. The function of TNFAIP3 mainly relies on its ubiquitin degradation to regulate intracellular protein expression [32]. Recent studies suggested that TNFAIP3 may have a vital role in the invasion and proliferation of lung cancer and gastric cancer [31, 33]. Our results revealed that the expression of TNFAIP3 was decreased in cancer samples but decreased more under P, indicating that P could reduce inflammation better than S.

In addition, local changes in tumors and their microenvironment exist even in the early stage of lung cancer, as shown by the high levels of molecular biomarkers related to local metabolic activity and inflammation in tumor tissue compared to surrounding lung tissue [34]. In our study, propofol and sevoflurane showed different effects on the inflammatory environment, and propofol inhibited the local inflammatory response more than sevoflurane. Some studies have pointed out that local inflammation is associated with negative long-term outcomes in lung cancer [35]. Enhanced local inflammation was associated with the upregulation of adhesion molecules activated by local inflammatory reactions [35]. Based on this, it is reasonable to speculate that choosing propofol over sevoflurane may be more beneficial for lung cancer patients from the perspective of the local tumor microenvironment and long-term prognosis. Although our results are encouraging, we should also be aware that should this benefit translate into a clinically significant effect on the long-term prognosis, more rigorous prospective studies will be needed to clarify this question.

However, in this study, there are several limitations: (1) without long-term follow-up, it is still uncertain whether P and S have any different effects in terms of the survival rate of lung cancer patients after surgery; (2) the sample size was relatively small, and only three patients’ NGS data in each anesthesia group were used. Although the sample size was small, the sequencing quality was good, and the DEG identified in the study were able to separate the different groups, indicating the accuracy and reliability of the DEG.

## Conclusions

In summary, our study investigated the effects of P and S during lung cancer resection at the RNA transcript level using RNA-seq and discovered that P and S mainly influenced inflammation-related pathways. This is consistent with previous clinical studies. However, the DEG identified in this study showed that P and S might have similar effects on certain genes but also different effects on other genes. Our findings reveal the complex role of anesthesia drugs in tumorigenesis.

## Supplementary Information

**Additional file 1: Table S1.** Primers used in qPCR.

## Data Availability

All data generated in this study are included in this published article and its supplementary information files. RNA-seq raw reads have been deposited into NCBI SRA database (PRJNA724914).
